# Baicalin hydrate inhibits cancer progression in nasopharyngeal carcinoma by affecting genome instability and splicing

**DOI:** 10.18632/oncotarget.22868

**Published:** 2017-12-04

**Authors:** Weiwei Lai, Jiantao Jia, Bin Yan, Yiqun Jiang, Ying Shi, Ling Chen, Chao Mao, Xiaoli Liu, Haosheng Tang, Menghui Gao, Ya Cao, Shuang Liu, Yongguang Tao

**Affiliations:** ^1^ Key Laboratory of Carcinogenesis and Cancer Invasion, Ministry of Education, Xiangya Hospital, Central South University, Changsha, Hunan, China; ^2^ Cancer Research Institute, Central South University, Changsha, Hunan, China; ^3^ Department of Thoracic Surgery, Second Xiangya Hospital, Central South University, Changsha, Hunan, China; ^4^ Changzhi Medical College, Changzhi, Shanxi, China; ^5^ Institute of Medical Sciences, Xiangya Hospital, Central South University, Changsha, Hunan, China

**Keywords:** baicalin hydrate, genome stability, splicing, DNA methylation, m6A RNA methylation

## Abstract

Baicalin hydrate (BH), a natural compound, has been investigated for many years because of its traditional medicinal properties. However, the anti-tumor activities of BH and its epigenetic role in NPC have not been elucidated. In this study, we identified that BH inhibits NPC cell growth *in vivo* and *in vitro* by inducing apoptosis and cell cycle arrest. BH epigenetically regulated genome instability by up-regulating the expression of satellite 2 (Sat2), alpha satellite (α-Sat), and major satellite (Major-Sat). BH also increased the level of IKKα, Suv39H1, and H3K9me3 and decreased LSH expression. Interestingly, BH promoted the splicing of Suv39H1 via the enhancement of m6A RNA methylation, rather than DNA methylation. Taken together, our results demonstrated that BH has an anti-tumor role in NPC and revealed a unique role of BH in genome instability and splicing in response to DNA damage.

## INTRODUCTION

Nasopharyngeal carcinoma (NPC) is an aggressive malignancy within the head and neck. The development of NPC is significantly associated with Epstein-Barr virus (EBV) infection as, patients with NPC have a clinically high rate of EBV infection [1, 2]. Because of its physiological location, radiotherapy is the main treatment for NPC patients [3, 4]. Moreover, EBV plays a key role in metastases, genome instability and the increased radio- and chemo-sensitivity in NPC [5–7]. A high degree of DNA methylation was observed in individual tumors [8]. Evidence shows that EBV-encoded proteinlatent membrane protein 1 (LMP1) induces squamous hyperplasia and inhibits squamous differentiation and the signaling JNK pathways through the activation of DNA methylation [9]. These observations imply that aberrant DNA methylation enforces NPC malignancy. However, the mechanism of epigenetic regulation in NPC remains to be elucidated.

Genome instability is one of the main characteristics in most cancers, including NPC, that is involved in maintaining normal physiological activities, cell proliferation and differentiation [10, 11]. Numerous processes are associated with genome instability, such as reprogramming energy metabolism, DNA damage repair, abnormal gene expression, etc.[12–15]. Genome instability mainly occurs during the early stage of cancer. It is thought to be that carcinogenesis is the continuation of genome instability [16, 17]. Moreover, genome instability is accompanied by abnormal repeat sequence expression. Microsatellites are repetitive DNA sequences that occupy 30% of the human genome [18]. Satellite 2 (Sat2), alpha satellite (α-Sat) and Major satellite (Major-Sat) are repeat sequences that are located in different places in the genome, and they are markers of genome instability [19–21]. Proper centrosome replication has an important role in the maintenance of genomic stability. Loss of IKKα, a subunit of the IKK complex, leads to the up-regulation of centrosome replication and cyclin D1, affecting cell cycle progression [22, 23]. HP1α belongs to the HP1 protein family. It is a non-histone chromatin-associated protein that binds to H3 when it is tri-methylated at lysine 9 (H3K9me3) and is crucial for the remodeling of heterochromatin in response to DNA damage [24, 25]. Suv39H1 (suppressor of variegation 3-9 homolog 1) is a histone methyltransferase. The methylation of Suv39H1 leads to a decrease in H3K9me3, resulting in the release of heterochromatin, which affects genomic stability [26]. In addition, recent studies have shown that WRN associates with the heterochromatin proteins Suv39H1 and HP1α to determine human aging [27]. Collectively, these findings suggest the importance of genome instability for tumor growth itself and Suv39H1 might play a key role in the cell cycle process and genomic stability.

LSH (lymphoid-specific helicase), one of the members of the ATPase-dependent chromatin remodeling protein family SWI/SNF, epigenetically silences the expression of repetitive sequence elements and promotes the phosphorylation level of H2AX [28–30]. The subunit Brm of SWI/SNF complexes facilitates alternative splicing by decreasing the elongation rate of RNA polymerase II (RNAP II)[31]. Moreover, splicing regulates most processes in cancer, such as chromatin remodeling, signal transduction and cell differentiation [32]. Amazingly, approximately 95% of tumor gene mutations are associated with alternative splicing [33]. SON, a large Ser/Arg (SR)-related protein, is a splicing co-factor that contributes to the efficient splicing of cell cycle regulators [34]. The down-regulation of SON leads to severe impairment of genome integrity resulting from inadequate RNA splicing of cell cycle-related genes. Recent evidence shows that SON regulates cell survival and the maintenance of pluripotency in human embryonic stem cells [35]. Taken together, a precise target splicing regulator may have a potential role in tumor therapy.

Baicalin hydrate (BH), also known as Huang Qin, a natural compound that has four phenolic hydroxyls, is widely used in traditional Chinese medicine and has antiviral, antibacterial and anti-inflammatory properties [36–38]. The tumor-inhibiting effect of BH has been reported for numerous tumor types [39–41]. However, the epigenetic effect and the antitumor activity of BH in NPC has not been well studied.

In this study, we studied how BH-induced changes modify tumor growth. We describe an epigenetic mechanism of BH in the regulation of splicing and its contribution to genome stability.

## RESULTS

### Antitumor activity of BH in nasopharyngeal carcinoma

**Figure 1:**
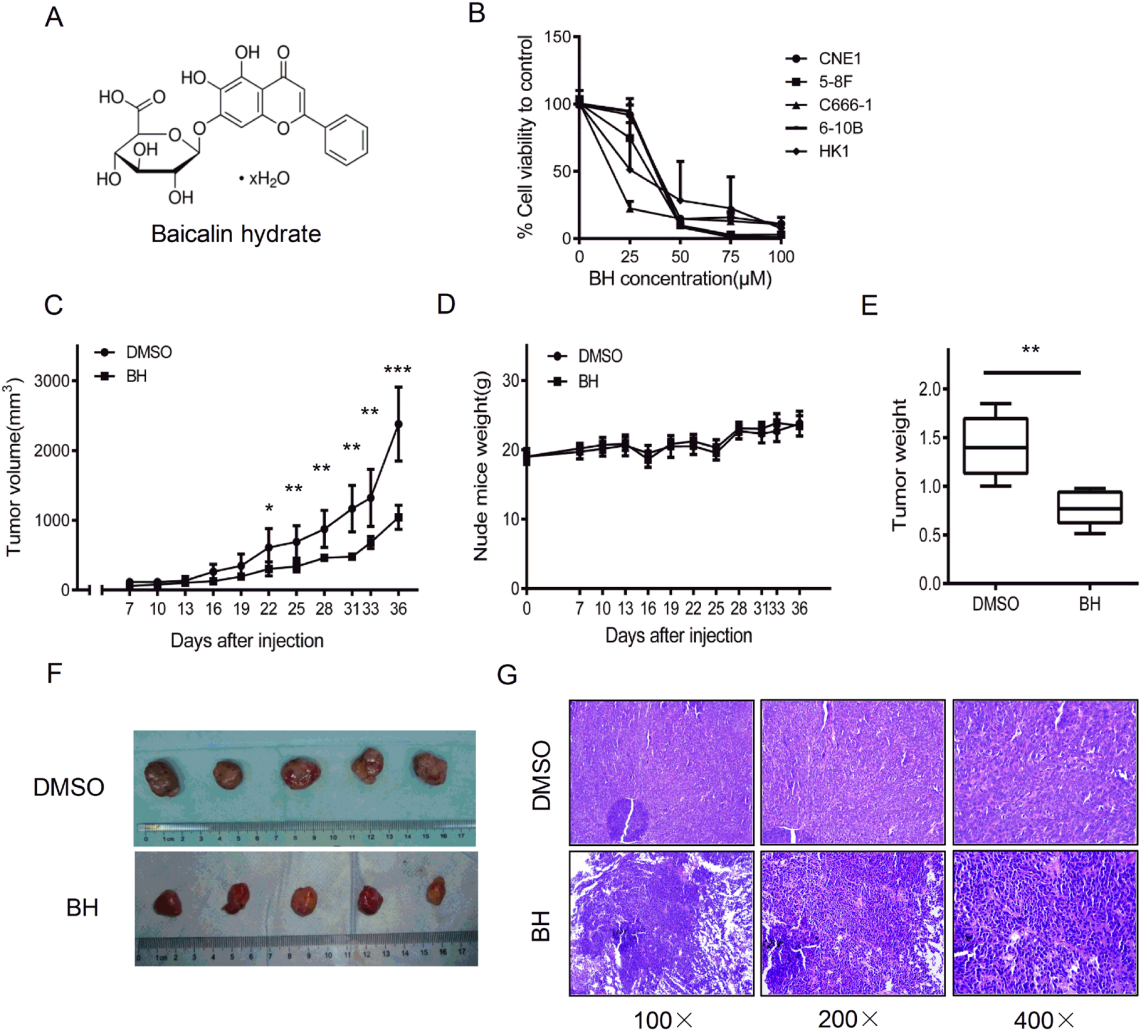
Baicalin hydrate reduced tumor growth *in vivo* and *in vitro* **(A)** The chemical structure of baicalin hydrate (C_21_H_18_O_11_ · xH_2_O MW: 446.36). **(B)** The cell proliferation of NPC cells was assessed after BH treatment. The data were normalized to the viability of the control group (DMSO-treated). The error bars represent the standard deviation (SD). **(C)** The tumor size of the xenograft, DMSO group and BH groups. Five independent experiments were measured for each group. The error bars represent the standard deviation (SD). ^*^P<0.05,^**^P<0.01, ^***^P<0.001. **(D)** The body weight of the nude mice in each group. Error Bars = ±SD. **(E)** Tumor weight. Error Bars = ±SD. ^**^ P<0.01. **(F)** The photographs of the tumors. Each group had 5 mice. **(G)** The tumor status was analyzed by H&E staining.

The chemical structure of baicalin hydrate (BH) is shown in Figure 1A. To evaluate the antitumor activity of BH in NPC, we performed an MTS assay to analyze the toxicity of BH in NPC cells. BH showed significantly cytotoxicity after five days (Figure 1B). We calculated the IC50 values of BH in different NPC cell lines using the SPSS 19.0 software. The IC50 values of different nasopharyngeal carcinoma cell lines were as follows: CNE1_IC50_=45.93 μM, HNE3_IC50_=23.17 μM, 5-8F_IC50_=32.89 μM, 6-10B_IC50_=34.03 μM, C666-1_IC50_=19.38 μM, and HK1_IC50_=31.22 μM. Next, we investigated the effect of BH on nasopharyngeal carcinoma cells *in vivo*. C666-1 cells were used in our experiment, as its IC50 value for BH is the smallest in these NPC cells. An *in vivo* nude mouse tumorigenicity assay was performed by injecting a total of 5×10^6^ C666-1 cells subcutaneously into the flank of 6–8 week old male nude mice. The chemical (10 mg/kg) was then administered to the mice intraperitoneally once every 3 days [42, 43] for 30 days for the course of treatment, and the tumor sizes were measured. As expected, BH significantly inhibited tumor growth. The average tumor volumes are shown in Figure 1C. The tumors in the control group increased from 60.22±33.67 to 2381.12±550.15 mm^3^, whereas the tumors in the BH-treated group varied from 50.22±28.72 to 1043.59±172.32 mm^3^. We also observed tumor weight both in the BH-treated group and the DMSO group (Figure 1E). The data showed that there was a significant reduced tumor weight in the BH-treated groups compared to the DMSO-treated groups (P<0.01). The average weight of the tumors from the control group was 1.41±0.31 g, whereas the tumors from the BH-treated group were 0.78±0.18 g. The solid tumors in the BH-treated group were smaller than the control group. An approximately 69.5% inhibition rate was observed in BH-treated group. However, the body weight did not change significantly in either group, and no evidence of drug-related toxicity was observed (Figure 1D). Photographs of the tumors are shown in Figure 1F, with an obvious reduction of tumor size observed in the BH group. H&E staining further demonstrate the anti-tumor activities of BH on NPC (Figure 1G). In the DMSO group, the heterogeneity and division of the tumors were obviously observed. However, in the BH-treated group, the nuclei decreased, the split phase was significantly decreased and necrosis occurred. These results suggest that BH significantly inhibit tumor growth *in vivo* and *in vitro*.

### Baicalin hydrate induces cell cycle arrest and apoptosis

**Figure 2:**
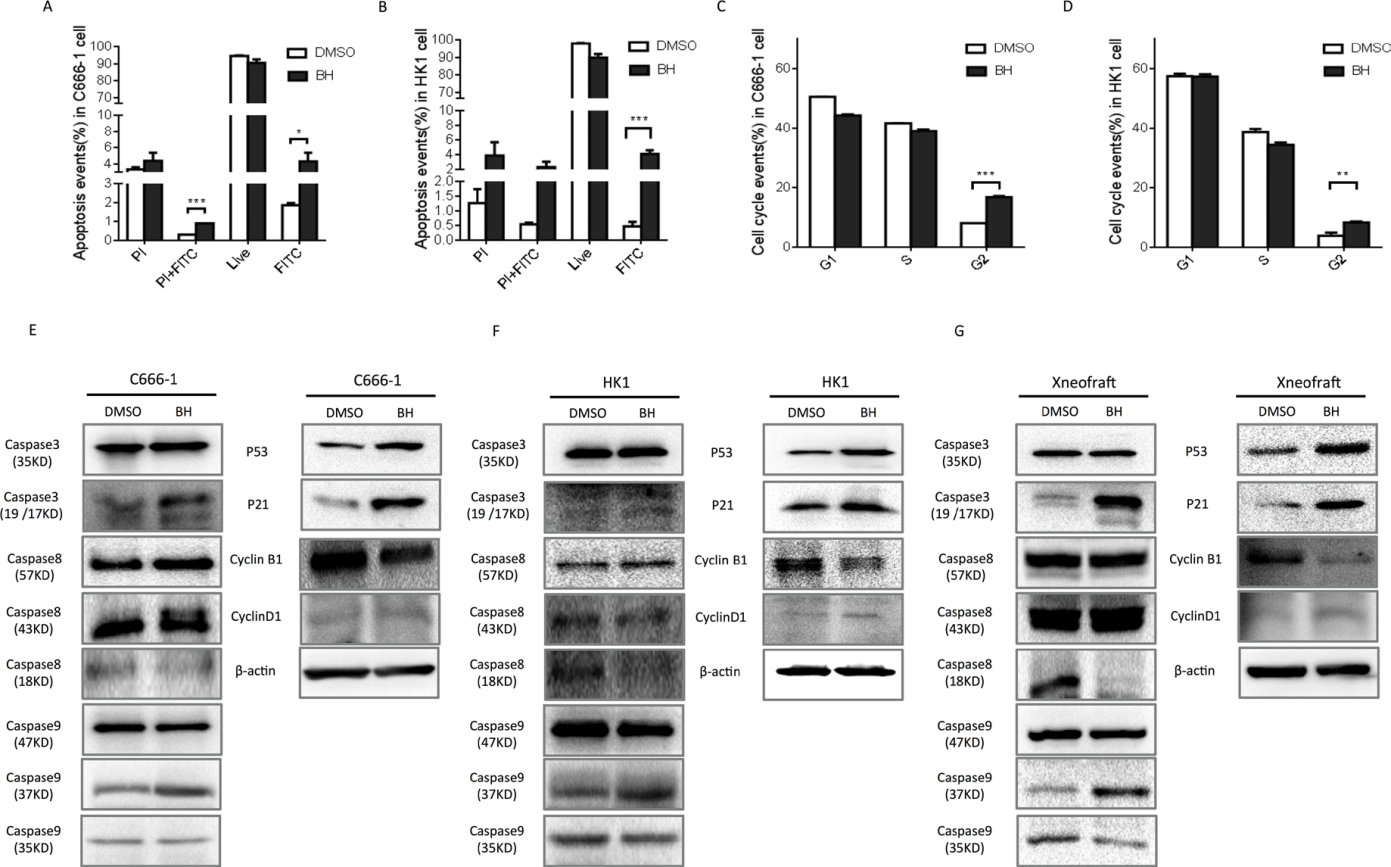
BH induced cell apoptosis and cell cycle arrest at the G2/M phase **(A)** The quantified results of annexin V/PI double staining in C666-1 cells treated with or without 19.38 μM BH for 5 days. The apoptotic events were calculated using the average of three independent experiments with similar results. Error Bars = ±SD, ^***^ P<0.001, ^*^ P<0.05. **(B)** The quantified results of the annexin V/PI double staining of HK1 cells treated with or without 31.22 μM BH for 5 days. Apoptotic events were calculated using the average of three independent experiments with similar results. Error Bars = ±SD, ^***^ P<0.001. **(C)** FACS analysis was used to detect the cell cycle progression of C666-1 cells after treatment with 19.38 μM BH or DMSO for five days. The data were shown as qualitative data. Error Bars = ±SD, ^***^ P<0.001. **(D)** The statistics of the FACS analysis of the HK1 cells after treatment with 31.22 μM BH or DMSO for five days. ^**^ P<0.01. The data shown are representative of three experiments showing similar results. **(E-G)** Western-Blot analysis was performed to assess the level of the indicated genes’ proteins. The xenograft represents proteins from the C666-1 solid tumor. The cells were treated with BH (HK1 cells 31.22 μM; C666-1 cells 19.38 μM) or DMSO for five days.

To understand the biological effects of BH in nasopharyngeal carcinoma, HK1 and C666-1 cells were treated with their IC50 value of BH for five days (HK1: 31.22 μM; C666-1: 19.38 μM). DMSO was used as a control (1:2000), and three independent experiments were done using flow cytometry. Flow cytometric analysis with annexin V/PI double staining were used to determine the levels of apoptosis induced by BH. We indeed observed a statistically significant level of apoptosis induced by BH (Figure 2A-2B). Notably, BH seems to mainly induce the early stage of apoptosis in NPC cells. We then observed the effects of BH on cell cycle arrest. Cells were treated with the IC50 values of BH for five days. There was no significant change in the S phase following BH treatment in our experiments. BH significantly induced cell cycle arrest at the G2/M phase both in C666-1 (P<0.001) and HK-1 (P<0.01) cells (Figure 2C-2D). Apoptosis is a vital biological process for organisms to maintain cellular homeostasis, which is regulated in a complex manner. As p53 is crucial for apoptosis [44], we further probed whether BH induced apoptosis in a p53-dependent manner. Our results showed that BH increased the level of p53 and p21, consistent with the activation of caspases (caspase3, 8, and 9) *in vivo* and *in vitro* (Figure 2E-2G). Cyclin-related proteins are multifunctional enzymes that can modify various protein substrates involved in the cell cycle [45]. We found that BH increased the levels of Cyclin D1 and decreased the expression of Cyclin B1 (Figure 2E-2G). Collectively, these results clearly indicate that BH significantly induced cell cycle arrest at the G2/M phase by affecting the cyclin-related proteins level and induced cell apoptosis in a p53-dependent manner *in vivo* and *in vitro*.

### BH affects genome instability

**Figure 3:**
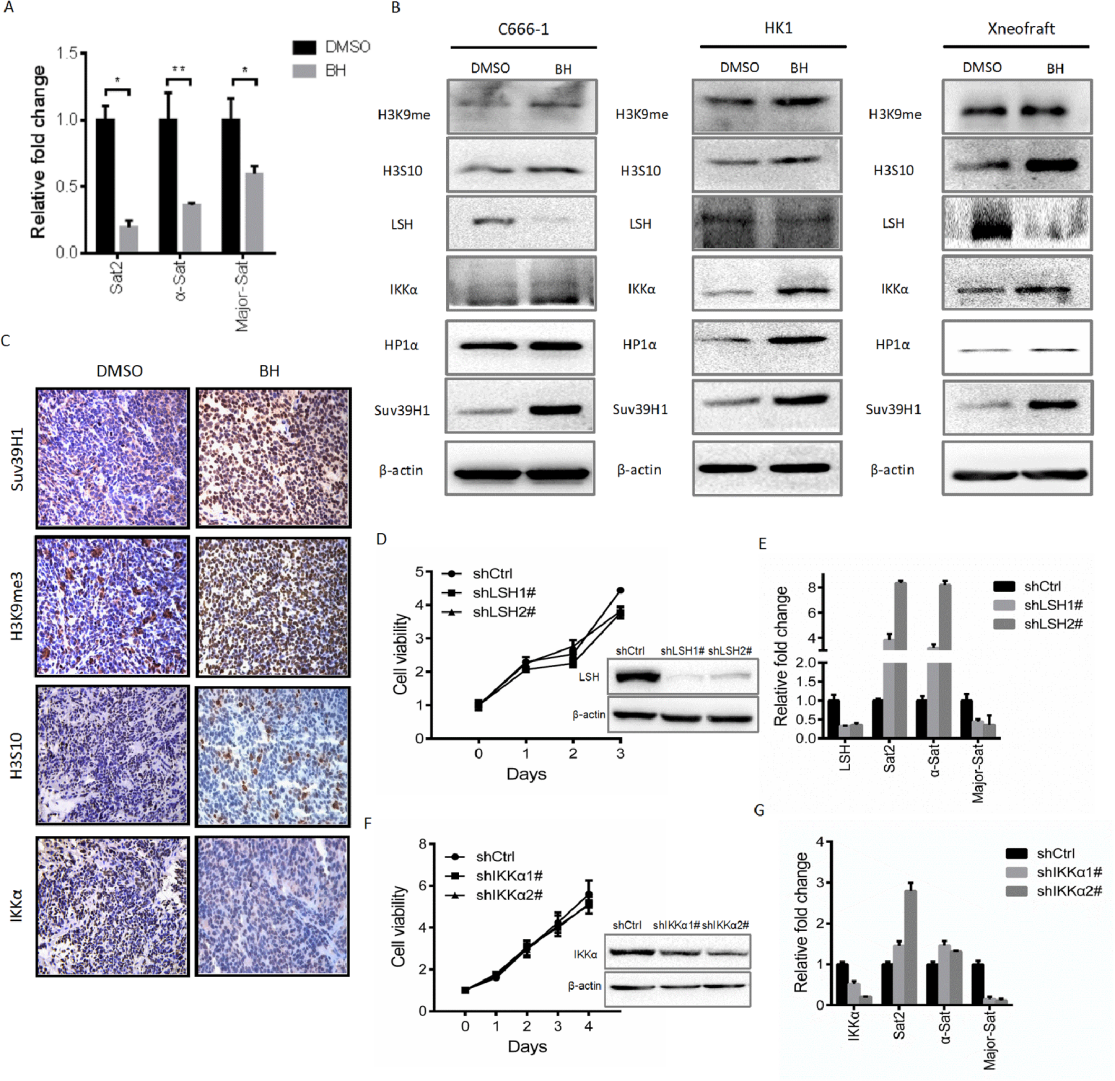
BH contributes to genome stability **(A)** C666-1 cells were treated with 19.38 μM BH for five days. The relative expression levels of Sat2, α-Sat and Major-Sat were measured by real-time PCR. The repeat sequences were significantly decreased. Sat _P_ =0.02789; α-Sat _P_ =0.005698; M-Sat _P_ =0.05505. ^*^P<0.05, ^**^P<0.01. The data are shown as the mean ± S.D (n=3). **(B)** The indicated proteins were examined by Western blot after treatment with BH for five days. The xenograft represents the proteins from C666-1 solid tumors. The cells were treated with BH (HK1 cells 31.22 μM; C666-1 cells 19.38 μM) or DMSO for five days. **(C)** Immunohistochemical analysis was used to examine the level of H3S10, Suv39H1, The levels of the H3K9me3 and IKKα proteins in xenografts. **(D)** The stable knockdown of LSH in C666-1 cells was performed. MTS assays were used for measurements. The level of LSH protein was detected by Western blot. ^**^P<0.01. **(E)** The mRNA expression of LSH and the indicated repeat sequences were analyzed by real-time PCR. The representative data from three independent experiments were expressed as the means ± S.D. ^*^P<0.05, ^***^P<0.001. **(F)** The stable knockdown of IKKα in HK1 cells was performed. MTS assays were used for measurements. The level of the IKKα protein was detected by Western blot. **(G)** The indicated genes in the stable knockdown of IKKα cells were measured by real-time PCR. The data are shown as the mean ± S.D (n=3). ^*^P<0.05,^**^P<0.01, ^***^P<0.001.

Based on the above observations, we hypothesized that in response to DNA damage, BH induced a structural change in heterochromatin by affecting genome instability. To verify this hypothesis, we first evaluated the transcripts of centromeric satellite repeats (Sat2, α-Sat, and Major-Sat) using real-time PCR after treating C666-1 cells with BH. The results are shown in Figure 3A. BH significantly decreased the mRNA expression level of Sat2 (P<0.05), α-Sat (P<0.001) and Major-Sat (P<0.05). When DNA damage occurred, H3K9me3 accumulated at the DNA damage site and facilitated the subsequent DNA damage repair. H3S10 phosphorylation is the major mitosis-specific phosphorylation of histone molecules and is important for chromosome condensation during mitosis in mammals [46, 47]. Suv39H1 is essential for active centromeres and directly or indirectly affects genome stability [48]. Here, we demonstrated that BH increased the level of tri-methylated H3K9, Suv39H1 and H3S10 *in vivo* and *in vitro* (Figure 3B). BH enhanced the production of HP1α (Figure 3B). These results suggest that BH is involved in the regulation of genome instability.

The activation of the NF-κB pathway is usually thought to promote cell survival. However, certain situations also activate NF-κB and induce pro-apoptotic gene transcription [49]. Recent work has uncovered that the disruption of ATP-dependent chromatin-remodeling complexes is a pivotal event in cancer pathogenesis [50, 51]. We found that BH increased the expression of IKKα *in vivo* and *in vitro* (Figure 3B and 3C). Our results also demonstrate that BH decreased the level of LSH (Figure 3B). To further assess whether the up-regulation of IKKα and the down-regulation of LSH, which were induced by BH, is associated with genome instability, we established a stable knock down of LSH in C666-1 cells and IKKα in HK1 cells using shRNA sequences. Our results showed that the knock down of LSH inhibited the proliferation of C666-1 cells and lead to the up-regulation of Sat2 and α-Sat. Conversely, the level of Major Sat decreased (Figure 3D-3E). Similarly, the knock down of IKKα suppressed the cell growth of HK1. We further examined the level of repeat sequences in these cells. Likewise, there was an obvious increase in the level of Sat2 and α-Sat, but there was a reduction in the level of Major Sat (Figure 3F-3G). These results suggest that blocking IKKα or LSH enhances the level of repeat sequences, except for Major Sat. Thus, these findings indicate that BH down-regulated the level of repeat sequences involved in the up-regulation of IKKα but not LSH.

### BH promotes alternative splicing through the up-regulation of m6A RNA methylation but not via DNA methylation

**Figure 4:**
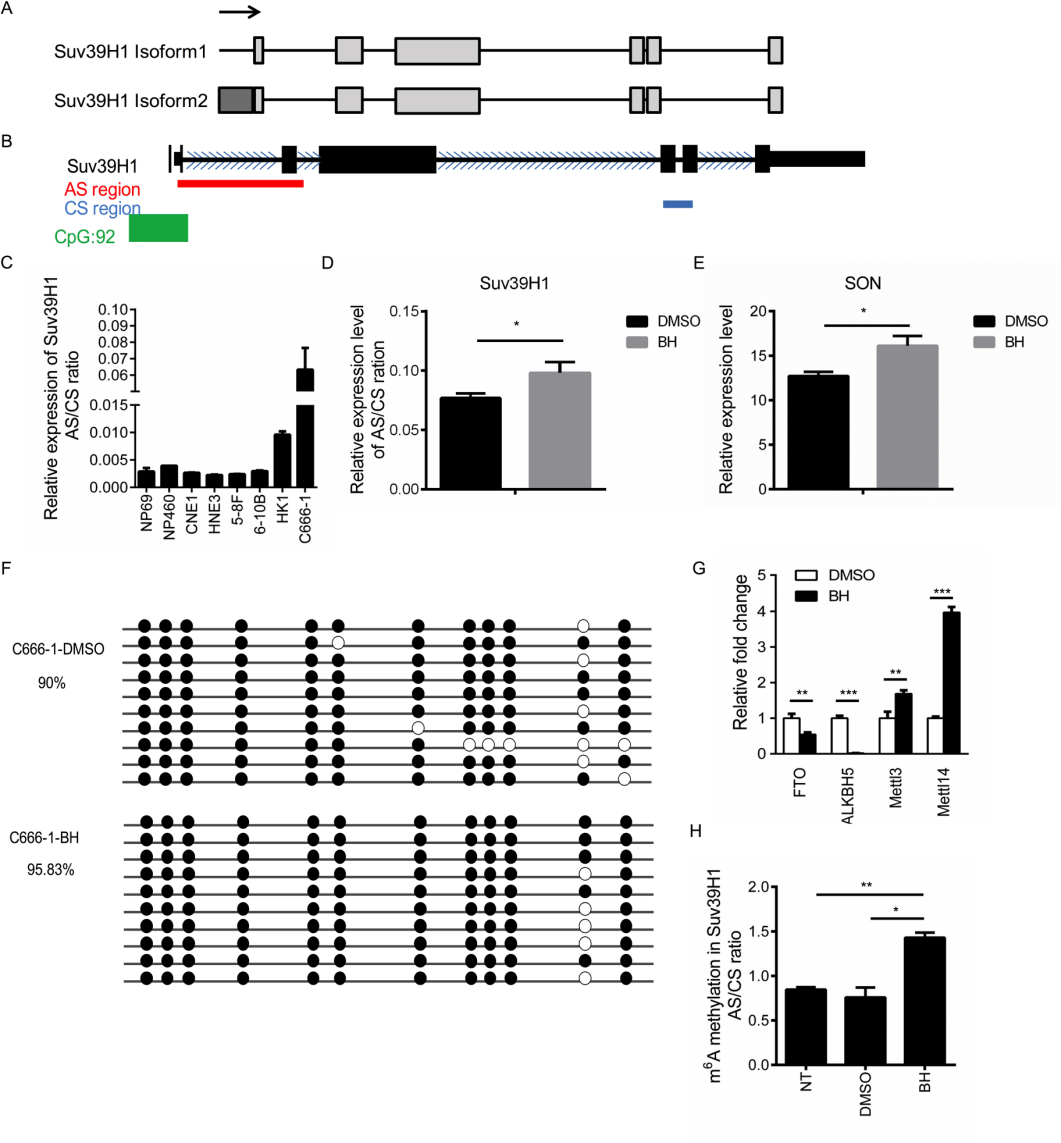
BH affects the splicing of Suv39H1 by up-regulated m6A RNA methylation **(A)** A schematic of the Suv39H1 isoforms according to AS-ALPS database. Light rectangles indicate exons, black lines indicate introns. Arrows indicate translation start sites. **(B)** A schematic of Suv39H1 according to the UCSC Genome Browser. Red lines represent the location of alternatively splicing primers; the blue line index shows the location of constitutively splicing primers. The green rectangle is CpG islands, and the blue arrows are the direction of transcription. **(C)** The quantification of the real-time PCR data of the AS/CS ratio of Suv39H1 mRNA in NPC cell lines. The mean of three independent measurements ± S.D are shown. **(D)** The quantification of the real-time PCR data of the AS/CS ratio of Suv39H1 mRNA showed that AS changed between the control and BH-treated group in C666-1 cells. The DMSO-treated group is the negative control. The mean of three independent measurements ± S.D are shown. ^*^P<0.05 **(E)** C666-1 cells were treated with BH or DMSO for five days. The mRNA expression level of SON was measured by real-time PCR. ^*^P<0.05 **(F)** A comparison of the level of DNA methylation after treatment with BH or DMSO in C666-1 cells. DNA methylation was determined using the bisulfite sequencing of the Suv39H1 exon 1 to exon 2 region. Each colored circle represents individual CpG cytosine methylation status within the analyzed region (black: methylated, white: unmethylated). The percentage of methylation was determined based on the number of methylated CpG cytosines divided by the total number of CpG cytosines within the analyzed sequences from all the PCR clones. **(G)** C666-1 cells were treated with BH for five days. The expression of the m6A RNA methylation-associated genes were detected by real-time PCR. The data are shown as the mean ± S.D. (n=3). FTO _P_ = 0.004576249; ALKBH5 _P_ = 1.81205E-05; Mettl3 _P_ = 0.004498575; Mettl14 _P_ = 5.75007E-06, ^**^P<0.01, ^***^P<0.001. **(H)** BH up-regulated the level of m6A RNA methylation. m6A was enriched within Suv39H1 mRNA. The quantification of m6A immunoprecipitation in the Suv39H1 gene was measured by using analyzed AS/CS ratio to represent the splicing events. The data are presented as the mean ±S.D, n = 3; BH vs DMSO P =0.005816; BH vs NT P =0.017163, NT: non-treatment, ^*^P<0.05, ^**^P<0.01.

Splicing prediction databases (AS-ALPS or ProSplicer database) indicated the existence of two isoforms of Suv39H1 (Figure 4A). The difference between these two isoforms is located in the promoter, which is approximately 11 amino acids. The splicing sites are located at the 5′ end of the gene. Primers were designed using the alternatively spliced (AS) splicing site and the constitutively spliced (CS) site (3′ end of the Suv39H1), and the ratio of these two amplicons (AS/CS ratio) were measured by real-time PCR. The location of these PCR products were modeled in Figure 4B. Our results showed that the AS/CS ratio of Suv39H1 was higher in C666-1 cells compared to the other NPC cells (Figure 4C). We then asked whether BH affects the alternative splicing of Suv39H1. Our results showed that BH obviously increased the the AS/CS ratio compared to that of the control group (Figure 4D). Moreover, BH increased the mRNA expression of SON (Figure 4E). These results provide evidence that BH affects the alternative splicing of Suv39H1.

DNA methylation promotes the pattern of splicing in exon inclusion and transcriptional elongation [52]. To further clarify the role of BH in alternative splicing regulation, we detected the DNA methylation of Suv39H1 through bisulfite sequencing-linked PCR. Unexpectedly, there were no significant changes in the level of DNA methylation with or without BH treatment (Figure 4F). RNA methylation in m6A is enriched at exonic splice sites and influences mRNA splicing [53]. Therefore, we examined the expression of m6A RNA methylation-associated methyltransferase at the mRNA level. Mettl3 and Mettl14 are two methyltransferases that methylate adenosine residues to form m6A in RNA molecules [54]. FTO and ALKBH5 are demethylases that remove the methyl group from m6A within RNA [55, 56]. Interestingly, we found that the treatment of C666-1 cells with BH significantly decreased the mRNA expression level of FTO (P<0.01) and ALKBH5 (P<0.001), while the levels of Mettl3 (P<0.01) and Mettl14 (P<0.001) were significantly up-regulated (Figure 4G). Furthermore, we performed MeRIP (methylated RNA immunoprecipitation) PCR to verify the influence of BH on the m6A RNA methylation in the AS/CS ratio of Suv39H1. Our results indicated that BH significantly up-regulated the level of m6A RNA methylation in Suv39H1 compared to DMSO group (P<0.05) and untreated group (P<0.01, Figure 4H). These results demonstrate that BH influenced the splicing of Suv39H1 through the up-regulation of m6A RNA methylation rather than DNA methylation.

## DISCUSSION

**Figure 5:**
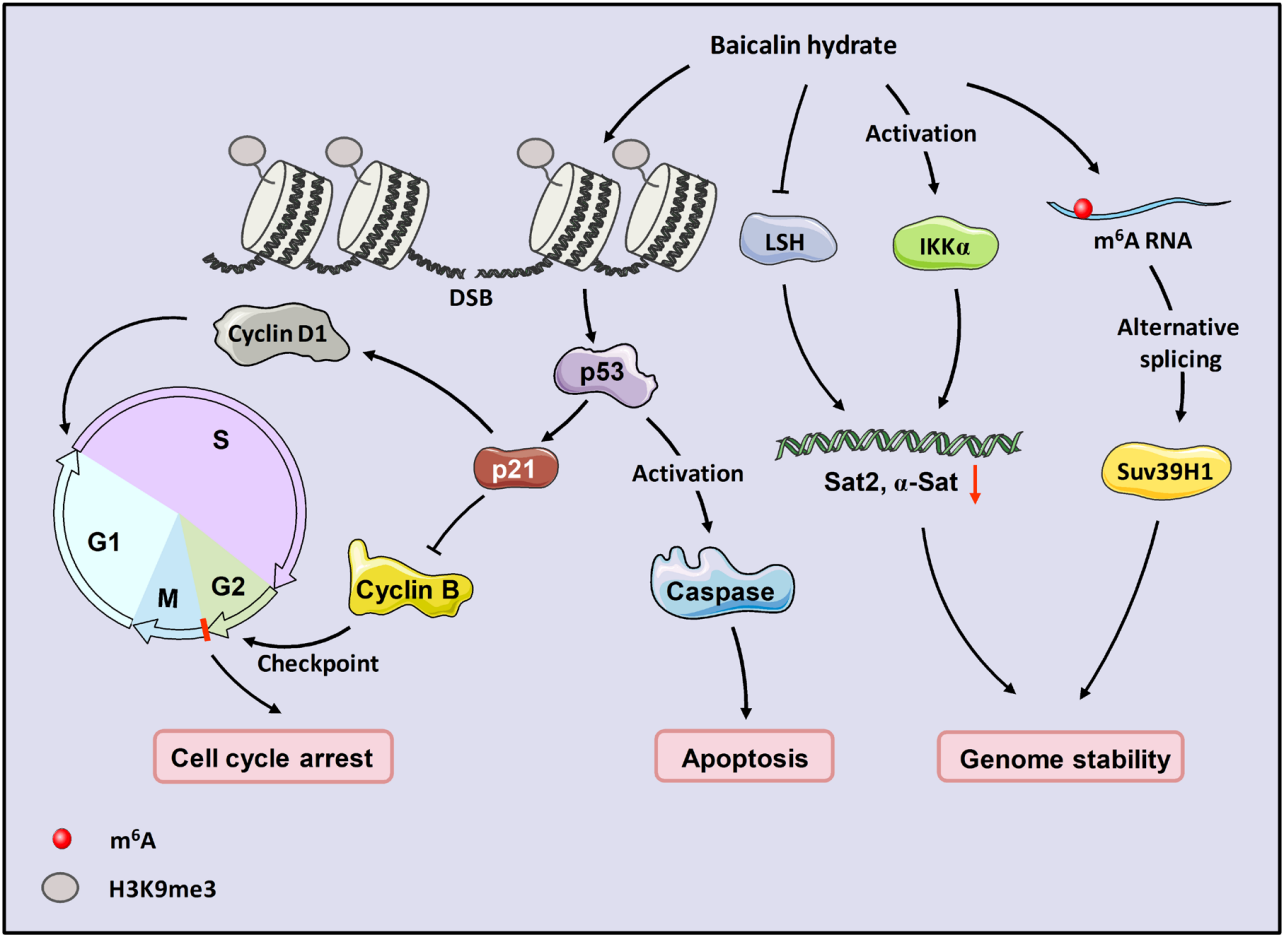
Model for proposed role of baicalin hydrate in NPC BH induced apoptosis in a p53-dependent manner. It also affected genome instability through the activation of IKKα, induced cell cycle arrest at the G2/M phase, and regulated the splicing of Suv39H1 through m6A RNA methylation.

In this study, we identified the antitumor effects of baicalin hydrate in nasopharyngeal carcinoma. We found that baicalin hydrate has a potential role in the maintenance of genome stability and alternative splicing. BH inhibited cell viability and tumor growth in NPC and induced early apoptosis and cell cycle arrest at the G2/M phase. In the presence of BH, the level of repeat sequences was decreased. The phosphorylation of H3S10 and tri-methylated H3K9 was up-regulated, indicating that BH induced DNA damage and epigenetic regulated genome instability. The activation of IKKα and the down-regulation of LSH were observed after the treatment of BH. Meanwhile, the loss of IKKα and LSH facilitates genome instability, resulting in the increased expression of repeat sequences. Moreover, BH increased the level of Suv39H1 and SON, promoted the splicing of Suv39H1 by activating m6A RNA methylation but did not change the DNA methylation of Suv39H1 (Figure 5).

Inducing apoptosis or cell cycle arrest in tumor cells is a common and efficient strategy for antitumor therapy. Evidence suggests that baicalin induces cell apoptosis in breast cancer and prostate cancer [39, 40]. Recent studies have reported that BH causes cell growth inhibition and cycle arrest in colorectal cancer [41], but there are fewer studies on its anti-tumor activity in NPC. To understand whether the BH-induced inhibition of cell viability was due to cell cycle arrest or cell apoptosis, FACS was performed. The data showed that BH induced cell apoptosis and cell cycle arrest at the G2/M phase (Figure 2). Cyclin B1 transcription begins at the end of S phase after DNA replication, and mitotic entry is determined by the level of active cyclin B1/CDK1 complex [57]. At entry into mitosis, cyclin B1-CDK1 promotes chromosome condensation, nuclear lamina breakdown and mitotic spindle assembly. Cyclin D1 is a critical target for proliferative signals in G1 phase. Aurora B is a member of the conserved protein kinases of the Aurora family, which mediates mitotic checkpoint functions during mitosis, especially at the G2/M checkpoint [58]. More importantly, Aurora B is primarily responsible for the phosphorylation of histone H3 serine 10 (H3S10), and H3S10 plays a key role in the super-condensation and super-compaction of chromosomes during mitosis [59]. In the G2 phase, the dephosphorylation of H3S10 and H3K9me3 at pericentric regions results in HP1α binding to these domains, and the location and appearance of HP1α relies on H3S10 phosphorylation [60, 61]. Although the level of Aurora B was not detected, we observed that BH up-regulated the phosphorylation of H3S10 and H3K9me and increased the expression level of HP1α, which is indispensable for proper heterochromatin formation and function (Figure 3).

LSH, also known as HELLS, belongs to the SWI/SNF family, ATP-dependent chromatin remodeling complexes that interact with γ-H2AX and modulate checkpoint activation, targeting the chromatin at DSBs (DNA double-strand breaks) [28, 62]. LSH was shown to cooperate with partners, such as G9a, to drive cancer progression [63–66]. Recent evidence has shown that LSH is required for DNA methylation at repeat sequences and mutations in LSH causes genome instability, resulting in the hypomethylation of Sat2 and α-Sat [67, 68]. LSH seems to be a driver in the process of tumor development [65, 69]. Generally, the aberrant overexpression of satellite repeats and Sat2 DNA hypomethylation are detected in a majority of cancer cells [70]. Major Satellite DNA, also known as gamma Sat, was flanked by α-Sat and initiates epigenetic silencing of pericentromeric genes [71]. The activation of the NF-κB pathway is common in NPC and may contribute to NPC development [23, 72, 73]. However, other studies have shown that the activation of the NF-κB pathway induced by natural components or chemicals suppresses tumor growth [17, 74]. The role of IKKα in NPC requires further investigation. Here, we reported that BH significantly decreased the level of repeat sequences, including Sat2, α-Sat and Major-Sat. Our findings further confirmed that BH up-regulated the expression of IKKα. The knock down of LSH and IKKα resulted in the up-regulation of repeat sequences, including Sat2 and α-Sat, while the level of Major-Sat was decreased, which might be related to its location at the distal centromere region. Evidence has shown that IKKα works as a negative centrosome duplication regulator, inhibits centrosome amplification, and maintains genome integrity [75]. We assumed that BH increased the IKKα levels and then regulated microsatellite markers to promote genome stability. While these effect of BH did not affect the distal centromere region, for there was no change in the mRNA expression on Major-Sat with or without the up-regulation of IKKα. Our research provides novel evidence for how IKKα may be involved in the maintenance of genome stability, which is induced by treatment with BH in NPC.

Transcript splicing is a key regulatory step for the proper expression of intron-containing genes. In human cancer cells, alternative splicing plays a key role in the development of cancer. Multiple studies have suggested that Suv39H1 epigenetically regulates the process of splicing and affects genome stability [26, 76, 77], whereas the splicing of Suv39H1 has not been extensively studied. Evidence has shown that DNA methylation levels affect the splicing pattern of exons and approximately 20% of the regulatory effect of DNA methylation on splicing can be explained by HP1 proteins [78–80]. Splicing factors also contribute to the process of alternative splicing [35]. Treatment with BH induced increasing levels of SON (a splicing co-factor) and promoted the splicing of Suv39H1; however, DNA methylation at the splice sites of Suv39H1 did not significantly change. Our previous study showed that BH reduced the level of HP1α, thus, directly or indirectly indicating that BH enhances the splicing of Suv39H1, regardless of the level of DNA methylation. Apart from DNA methylation, recent evidence has demonstrated that the increasing level of m6A RNA methylation leads to the splicing pattern of exon inclusion [81]. Here, we observed that the increasing level of m6A RNA methylation at the splice sites of Suv39H1 by BH relies on Mettl3 and Mettl14. Additionally, BH reduced the demethylase of m6A. Taken together, our results demonstrate that BH promotes the alternative splicing independent of DNA methylation by increasing the level of m6A RNA methylation.

In a summary, our study provides a possible epigenetic regulation mechanism for the observed ability of BH to stimulate cancer cell death and identified a novel way that BH affects genome instability and alternative splicing. Further research is needed to investigate the function of different Suv39H1 isoforms and the relationship between Suv39H1 and genome stability in BH-treated cells.

## MATERIALS AND METHODS

### Cell cycle assay

BH- and DMSO-treated C666-1 and HK1 (5×10^3^) tumor cells were harvested after five days of culture under normoxic conditions. The cells were harvested and fixed in 75% ethanol at -20°C overnight. The cells were stained with propidium iodide (PI) solution at a final concentration of 50 μg/ml containing 50 mg/ml RNase A. Cell cycle data were acquired and analyzed using an Accuri C6 machine.

### Real-time RT–PCR

The total RNA was extracted using TRIzol reagent (Invitrogen). The RNA was used to generate cDNA using SuperScript III RT (Invitrogen) with an oligo-dT primer. Real-time PCR was performed using Q SYBR Green Supermix from Bio-Rad following instructions recommended by the manufacturer. Actin was used as a control, primers were listed in Table 1.

**Table 1:** Primers of indicated genes

Gene	DNA accession number	Forward primer ( 5’→ 3’)	Reverse primer ( 5’→ 3’)	Product size
β-actin	NM_001101	AGAGCTACGAGCTGCCTGAC	AGCACTGTGTTGGCGTACA	184bp
Suv39H1 (AS)	NM_001282166	GGAGACTGACTTGACCAATGG	TAGAGATACCGAGGGCAGGG	159bp
Suv39H1 (CS)	NM_003173	CCCTGCCCTCGGTATCTCTA	CACTTGAGATTCTGCCGTGG	156bp
Sat2	X72623	CATCGAATGGAAATGAAAGGAGTC	ACCATTGGATGATTGCAGTCAA	160bp
α-Sat	M38467	CTGCACTACCTGAAGAGGAC	GATGGTTCAACACTCTTACA	139bp
Major-Sat	NM_002970	GACGACTTGAAAAATGACGAAATC	CATATTCCAGGTCCTTCAGTGTGC	74bp
IKKα	NM_001278	ATGAAGAAGTTGAACCATGCCA	CCTCCAGAACAGTATTCCATTGC	110bp
LSH	NM_001289074	AGAAGGCATGGAATGGCTTAGG	GCCACAGACAAGAAAAGGTCC	151bp
METTL3	NM_019852	CATTGCCCACTGATGCTGTG	AGGCTTTCTACCCCATCTTGA	82bp
METTL14	NM_020961	GAGTGTGTTTACGAAAATGGGGT	CCGTCTGTGCTACGCTTCA	172bp
FTO	NM_001080432	ACTTGGCTCCCTTATCTGACC	TGTGCAGTGTGAGAAAGGCTT	145bp
ALKBH5	NM_017758	ATGCACCCCGGTTGGAAAC	GACTTGCGCCAGTAGTTCTCA	250bp

### Establishment of stable knock down cells using short-hairpin RNA

The knockdown of genes was performed with specific shRNAs delivered by a lentiviral system purchased from Addgene. In brief, to generate the lentivirus containing the specific shRNA, 293T cells were co-transfected with 1.5 μg of pMD2.G, 4.5 μg of psPAX2 compatible packaging plasmids and 6 μg of plasmid bearing the specific shRNA for 7 h. Then, the cells were incubated with fresh medium (with serum) at 37°C in a humidified chamber containing 5% CO_2_ to produce the lentivirus. The culture medium containing lentivirus was collected and stored at 80C as aliquots for further use. To deliver the specific shRNA construct, the cells were infected with the lentivirus bearing specific shRNA in growth medium containing 8 mg/ml (1:1000) polybrene and incubated at 37°C. Afterwards, C666-1 and HK1 cells (transfected with shLSH plasmids (PLKO.1) and shIKKα plasmids (GV115, purchased from Genechem) with deficient puromycin resistance) were subcultured and treated with 0.5 μg/ml and 1 μg/ml puromycin, respectively. The shRNA constructs targeting the gene of interest refer to the sequences as follows: sh-LSH#1: (TRCN030767R20310) ATTAGACGGCACTTCATATTC; sh-LSH#2 (it is a pool lentivirus contain TRCN030767R20308, TRCN030767R20309 TRCN030767R20310, TRCN030767R20306, TRCN030767R20307) TRCN030767R20308: AACAAGGCGATAAACAACAAC; TRCN030767R20309 AATTGTTTCTTTCTCACTGGA; TRCN030767R20306 TTCTACAGGGATATTCACTTC; TRCN030767R20307 AAGTCATCAAATACATCTGGC. Sh-IKKα#1: (Genechem ID: 25460-1) GCAAATGAGGAACAGGGCAAT. Sh-IKKα#2: (Genechem ID: 25464-1) GGTTAATGTAGTATGGTATAT.

The scrambled shRNA vector SHC002 (shLSH-Ctrl) (Insert Sequence: 5’-CCGGCAACAAGATGAAGAGCACCAACTCGAGTTGGTGCTCTTCATCTTGTTGTTTTT-3’) was used as a control. shIKKα-Ctrl was purchased from Genechem (GV115, CCTATTTCCCATGATTCCTTCATA).

### Methylated RNA immunoprecipitation-PCR

C666-1 cells were treated with BH for five days, and a DMSO group was used as a control. The total RNA (approximately 300 μg/sample of total RNA was obtained for further application) was extracted using TRIzol reagent (Invitrogen). The RNA was then fragmented into 200 nt-sized fragments using NEB Next, Magnesium RNA Fragmentation Module (Cat. no. E6150S, NEB) according to the manufacturer’s instructions, and then, the fragments were subjected to three rounds of m6A immunoprecipitation (15 μg of m6A antibody was used in each round). The fragmented RNA that did not bind to the m6A antibody was used as input (5 μg)[82]. Next, we performed RT-PCR as mentioned previously to analyze the m6A RNA methylation level.

### Nude mice and study approval

Female nude mice (5-weeks-old) were used to establish xenografts by subcutaneous injection of *in vitro*-cultured C666-1 cells (5×10^6^ cells/100 μl) into the flanks of donor nude mice. After the injection of the tumors, the mice were randomized into two groups (DMSO group and BH group), each containing five mice (two tumors/mouse). After one week, the animals were treated orally with BH (10 mg/kg) once every three days for 30 days. When the tumors reached approximately 1000 mm^3^ in size, the mice were anesthetized with diethyl ether, and the tumor masses were surgically removed from the mice. The control mice received an equal volume of a physiological saline mixture (DMSO:physiological saline of 1:2000). The tumors were measured in two diameters with calipers to calculate the tumor volume using the formula: V =1/2^*^D^*^d^2^, where D and d are the larger and smaller diameters, respectively. The statistical significance of differences in tumor volume, wet tumor weight and body weight between the control and treated mice were assessed by Student’s t-test.

All the procedures for animal studies were approved by the Institutional Animal Care and Use Committee of the Central South University of Xiangya School of Medicine and conform to the legal mandates and federal guidelines for the care and maintenance of laboratory animals.

### Statistical analysis

The experiments were repeated at least three times. The results are expressed as the mean ± SD or SEM as indicated. A 2-tailed Student’s t test was used for intergroup comparisons. A p value less than 0.05 was considered statistically significant (^*^p < 0.05, ^**^ p <0.01, ^***^ p <0.001).
